# Integration of Functional Materials in Photonic and Optoelectronic Technologies for Advanced Medical Diagnostics

**DOI:** 10.3390/bios15010038

**Published:** 2025-01-10

**Authors:** Naveen Thanjavur, Laxmi Bugude, Young-Joon Kim

**Affiliations:** 1 Department of Electronic Engineering, Gachon University, Seongnam 13120, Republic of Korea; naveenthanjavur@gachon.ac.kr; 2 Department of Semiconductor Engineering, Gachon University, Seongnam 13120, Republic of Korea; 3 Dr. Buddolla’s Institute of Life Sciences, A Unit of Dr. Buddolla’s Research and Educational Society, Tirupati 517506, India

**Keywords:** photonic devices, optoelectronic devices, functional materials, medical diagnostics, advanced imaging technologies

## Abstract

Integrating functional materials with photonic and optoelectronic technologies has revolutionized medical diagnostics, enhancing imaging and sensing capabilities. This review provides a comprehensive overview of recent innovations in functional materials, such as quantum dots, perovskites, plasmonic nanomaterials, and organic semiconductors, which have been instrumental in the development of diagnostic devices characterized by high sensitivity, specificity, and resolution. Their unique optical properties enable real-time monitoring of biological processes, advancing early disease detection and personalized treatment. However, challenges such as material stability, reproducibility, scalability, and environmental sustainability remain critical barriers to their clinical translation. Breakthroughs such as green synthesis, continuous flow production, and advanced surface engineering are addressing these limitations, paving the way for next-generation diagnostic tools. This article highlights the transformative potential of interdisciplinary research in overcoming these challenges and emphasizes the importance of sustainable and scalable strategies for harnessing functional materials in medical diagnostics. The ultimate goal is to inspire further innovation in the field, enabling the creation of practical, cost-effective, and environmentally friendly diagnostic solutions.

## 1. Introduction

**Figure 1:**
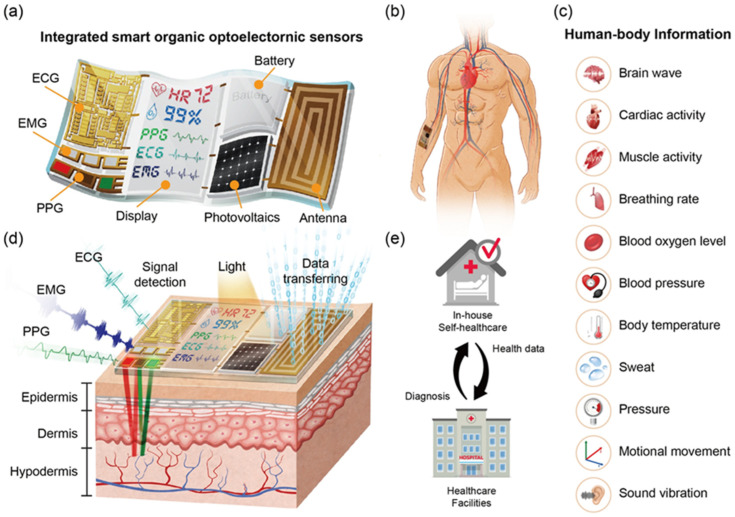
Illustration of key properties and biomedical imaging applications. (**a**) Stretchable organic optoelectronic devices. (**b**) A smart sensing system placed on the skin can detect artery and vein impulses. (**c**) Smart sensing devices are being used to read information from the human body. (**d**) Smart sensing devices can be seamlessly incorporated into the human body, facilitating real-time signal capture and data transmission through wireless networks. (**e**) The combination of wireless data transmission technologies and smart sensing systems is critical for developing human-friendly electronics and diagnostic medical applications that rely on timely feedback from healthcare facilities. These advancements make optoelectronic devices indispensable in applications like high-resolution imaging and real-time therapeutic monitoring. Reproduced from ref. [8] with permission from Elsevier Copyright 2021.

The integration of functional materials with photonic and optoelectronic devices has catalyzed notable progress in the field of medical diagnostics. Traditional diagnostic materials and technologies, while foundational, often fall short of meeting modern healthcare demands. For example, conventional organic dyes used in imaging are prone to photobleaching, limiting their use in long-term monitoring applications. Similarly, silicon-based materials in photonic devices lack the flexibility and biocompatibility necessary for wearable and implantable devices. These limitations underscore the need for advanced functional materials, such as quantum dots (QDs) and perovskites, which offer improved photostability, tunable properties, and enhanced compatibility with next-generation diagnostic platforms [1,2]. These advancements are crucial for the early detection of diseases and the implementation of personalized medicine approaches, ultimately enhancing patient outcomes and healthcare efficiency [3]. Functional materials comprise a diverse array of substances, each contributing distinct advantages to the operational capabilities of photonic and optoelectronic devices. Among these, low-dimensional van der Waals materials have emerged as powerful candidates for photonic and optoelectronic medical diagnostics. These materials, such as transition metal dichalcogenides (TMDs) and black phosphorus, exhibit exceptional transport and optical properties, including high carrier mobility, strong light–matter interaction, and tunable bandgaps, making them suitable for applications like high-resolution imaging and biosensing [4]. For instance, QDs exhibit size-tunable fluorescence, enabling flexible imaging options across various biological systems [5]. Additionally, optoelectronic devices possess multiple advantageous properties, such as being wearable, ultra-lightweight, implantable, stretchable, and disposable, which minimize the discomfort on human tissues and skin interfaces [6]. Wearable sensing systems enable real-time monitoring of physiological signals, including heart rate and respiration. These systems utilize advanced photoplethysmography (PPG) or impedance pneumography techniques to measure blood flow and respiratory movements, respectively. For instance, recent advancements [7] have demonstrated wearable sensors capable of accurately detecting heart rate variability and respiratory patterns under dynamic conditions, making them essential for non-invasive health monitoring. Wireless data transmission integrated with advanced sensors is vital for medical diagnostics and user-friendly healthcare electronics (Figure 1).

Perovskites, known for their exceptional light absorption and high charge carrier mobility, have emerged as promising candidates for next-generation optoelectronic applications. Meanwhile, plasmonic nanomaterials leverage surface plasmon resonance to significantly enhance signal detection, rendering them ideal for applications in biosensing [2,9]. Organic semiconductors, recognized for their ease of processing and tunable electronic properties, open new opportunities for fabricating diagnostic devices that are flexible and lightweight [10]. Integrating these advanced materials into diagnostic technologies presents several challenges. Material stability, scalability, and reproducibility are critical factors that ensure the clinical viability of devices. For instance, advanced quantum-dot materials are widely utilized in sensing applications; however, their diminutive size (5–20 nm) introduces significant limitations, complicating their centrifugation using conventional systems. Additionally, the clinical translation and optimization of nanomaterial designs, along with necessary chemical modifications, often require supplemental ultraviolet (UV) light sources to accurately measure fluorescent signals [11,12]. Furthermore, the sustainability of the materials employed and their environmental impact during production and disposal must be considered as part of the developmental process.

In recent years, substantial research efforts have concentrated on overcoming these hurdles, resulting in notable breakthroughs in nanotechnology, materials science, and biophotonics [13]. These advancements not only enhance the performance of diagnostic devices but also promote interdisciplinary collaboration among scientists, engineers, and healthcare practitioners to foster the development of novel applications. This review systematically explores recent innovations in functional materials, specifically highlighting their integration into photonic and optoelectronic devices for medical diagnostics. By elucidating how these materials enhance diagnostic capabilities, this article provides insights into the technical challenges and future research directions necessary for optimizing their practical applications in healthcare. The ultimate aim is to contribute to the ongoing discourse regarding the transformative potential of advanced imaging and sensing technologies in enhancing human health.

## 2. Quantum Dots in Photonic Devices

QDs have emerged as vital components in photonic devices, offering unique optical and electronic properties that significantly enhance the performance of various applications. Their ability to emit light at specific wavelengths and their nanoscale size provide substantial advantages across multiple fields, including telecommunications, biomedical imaging, and quantum computing.

### 2.1. Optical Properties of QDs

Various nanoparticle platforms, both organic and inorganic, have been extensively investigated for biomedical applications. Among these, inorganic nanoparticles, specifically QDs, are notable for their unique optical characteristics. They feature broad absorption spectra, high quantum yields, sharp emission peaks, excellent photostability, and size-dependent tunable emission [14]. In addition to QDs, superparamagnetic iron oxide nanoparticles (SPIO) and other metal oxides represent another class of inorganic nanoparticles used in biological imaging applications. A formulation of SPIO has been approved by the Food and Drug Administration (FDA) for clinical use, and iron oxide nanoparticles have been widely employed as MRI contrast agents [15]. QDs, as nanoscale semiconductor particles, exhibit remarkable optical properties, particularly their size-tunable emission wavelengths and high quantum yields.

Heavy metal-free biocompatible QDs with high photoluminescence quantum yields have enabled lymph node mapping in ex vivo imaging. Additionally, an innovative approach has been developed involving gadolinium-doped carbon dots for fluorescence/magnetic resonance imaging (MRI) [16]. Core–shell QDs, such as Cadmium Selenide Zinc Sulfide (CdSe@ZnS), have been utilized to quantitatively determine the extracellular nuclease activity of Staphylococcus aureus, employing QDs conjugated to a single-stranded Deoxyribose Nucleic Acid (ssDNA) fluorescent dye Fluorescence Resonance Energy Transfer (FRET) probe. Furthermore, multiphoton imaging of tumor biomarkers can be conducted by conjugating single-domain antibodies to CdSe@ZnS QDs, providing a viable alternative to conventional organic fluorophores for imaging biomarkers [17].

The emission wavelength of QDs can be precisely controlled by adjusting their size, allowing researchers to design QDs that emit specific colors ranging from ultraviolet to infrared [18,19]. This tunability arises from quantum confinement effects, which create discrete energy levels within the semiconductor material. Moreover, QDs demonstrate significantly higher photostability and brightness compared to traditional organic dyes, which are often prone to photobleaching during prolonged exposure to light [20,21]. These characteristics provide a robust platform for high-resolution imaging applications that require long-term observation of dynamic biological processes. The brightness and narrow emission spectra of QDs facilitate enhanced signal detection amidst complex biological backgrounds, rendering them invaluable tools for advancements in photonic devices used in medical diagnostics [22].

### 2.2. Application in Medical Imaging

**Figure 2:**
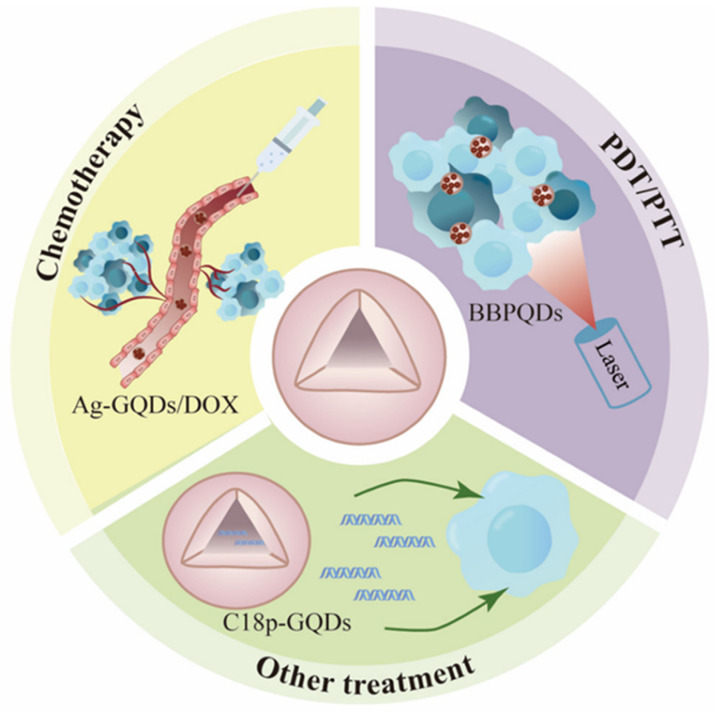
Depiction of non-functional QDs in targeted cancer therapies. Demonstrates how QDs selectively bind to tumor sites, enhancing imaging contrast and enabling precise localized therapy. These properties contribute to improved tumor identification and treatment efficacy, underscoring the potential of QDs in advanced diagnostic and therapeutic applications. Reproduced from ref. [22].

The integration of QDs into fluorescence imaging systems has revolutionized medical diagnostic techniques, enabling real-time monitoring of biological processes at the cellular and molecular levels [23]. QDs, in particular, have advantages in multiplexed imaging applications, where multiple biomarkers can be detected and analyzed simultaneously. Additionally, graphitic carbon nitride (C_3_N_4_) and indium phosphide (InP) are two non-toxic QDs well-suited for bioimaging due to their flexible fluorescence intensity [24,25]. This capability allows for comprehensive evaluation of disease states, as several physiological markers can be tracked within the same biological sample. For instance, in vivo imaging with QDs can provide critical insights into disease progression and treatment responses, offering a dynamic view of biological processes. Their multifunctional nature facilitates both imaging and therapeutic interventions, making QDs suitable candidates for the development of advanced theranostics platforms that streamline the diagnosis and treatment of diseases such as cancer, as shown in Figure 2. Consequently, the application of QDs in fluorescence imaging not only enhances the sensitivity and specificity of diagnostic tools but also opens avenues for innovative diagnostic strategies in personalized medicine.

### 2.3. Barriers and Emerging Prospects

A major regulatory hurdle for cadmium-based QDs lies in their inherent toxicity and environmental risks. Cadmium is classified as a carcinogen, and its use in medical devices poses significant challenges for obtaining regulatory approval, particularly in regions with strict environmental and safety standards, such as the European Union under the Restriction of Hazardous Substances (RoHS) Directive. Additionally, the disposal of cadmium-based QDs raises environmental concerns, as improper handling can lead to soil and water contamination. To overcome these challenges, researchers are developing heavy metal-free QDs, such as indium phosphide systems, which reduce toxicity without compromising optical performance. Furthermore, advancements in green synthesis methods, employing plant-based reducing agents or low-energy production processes, are being explored to minimize the environmental footprint of QD manufacturing. Regulatory frameworks are increasingly encouraging such sustainable innovations to balance technological progress with environmental stewardship and public safety [26]. Emerging non-toxic alternatives, such as carbon-based QDs (C-QDs), are gaining attention as viable substitutes for cadmium-based QDs due to their superior biocompatibility, environmental safety, and cost-effectiveness. These materials are derived from abundant and renewable sources, such as biomass, and exhibit excellent photostability and tunable fluorescence. For instance, recent studies have demonstrated the use of gadolinium-doped carbon dots for combined fluorescence and MRI, providing dual-modality imaging capabilities with minimal toxicity. Nitrogen- and sulfur-doped C-QDs exhibit improved quantum yields and stability, making them ideal for biosensing and bioimaging. These advancements highlight the potential of C-QDs in theranostics platforms, offering a safer and more sustainable approach to advanced diagnostic technologies. Continued research into surface functionalization and doping strategies is expected to further expand their applicability in medical diagnostics [27]. In recent years, various carbon-based quantum dots (C-QDs) have been synthesized with remarkable quality, non-toxic nature, bright luminescence, and excellent performance. Notable examples include C_3_N_4_ quantum dots (CNQDs), MXene quantum dots (MQDs), and graphdiyne quantum dots (GDQDs), which exhibit superior optical properties, excellent water solubility, and high stability as photocatalysts, making them highly advantageous for applications in image sensing and biomedical fields [28,29].

The future of QDs in medical diagnostics is poised for groundbreaking improvements, especially with the development of QD-based theranostics. This innovative approach has the potential to merge diagnostic imaging and therapeutic functionalities into a single platform, enabling the simultaneous detection and treatment of diseases. By harnessing the properties of QDs, researchers aim to create multifunctional agents that facilitate real-time disease monitoring and targeted drug delivery, ultimately improving patient management and treatment outcomes [30].

## 3. Perovskite Materials in Optoelectronic Devices

Perovskite materials exhibit high light-absorption coefficients, long charge carrier diffusion lengths, and intense photoluminescence, which make them exceptionally suitable for various optoelectronic devices [31]. The ease of fabrication and low-cost processing of these materials contribute to their excellent performance in applications such as solar cells and light-emitting diodes (LEDs). Furthermore, their inherent tolerance to defects enhances their functionality in device applications [32].

### 3.1. Properties and Advantages of Perovskites

Perovskite materials have garnered substantial attention in the field of optoelectronics due to their remarkable optoelectronic properties. A notable characteristic is their high absorption coefficients, which facilitate effective light harvesting, a critical factor for increasing the efficiency of devices such as solar cells and photodetectors [33]. These materials feature tunable bandgaps, allowing for customized optical properties that enable their use across a broad range of wavelengths tailored to specific applications. Such tunability, alongside low defect density, intense photoluminescence, suitable bandgap, strong carrier mobility, and high ion conductivity, positions perovskites as excellent candidates for high-performance optoelectronic devices, including photodetectors and LEDs [34,35,36]. Perovskite solar cells have reported certified efficiencies exceeding 22%, establishing them as leading technologies in the solar energy landscape. The versatility of perovskite materials facilitates their integration into a wide array of optoelectronic devices, including solar cells, LEDs, and photodetectors. This positions perovskites as key players in the transition towards affordable and sustainable energy solutions, particularly in solar energy applications, due to their relative abundance and low-cost fabrication methods [37]. Additionally, the development of perovskite materials is making significant inroads into medical imaging applications, such as X-ray detectors, thereby enhancing diagnostic precision [38].

### 3.2. Sensor Development and Applications

Perovskite photodetectors demonstrate exceptional sensitivity, detecting low-intensity light signals with high precision [39]. Tin-based perovskite materials, in particular, have emerged as promising lead-free alternatives in thin-film photodetectors (PDs) for various applications, including optical communications, night vision, and biomedical near-infrared imaging systems [40]. This sensitivity is particularly beneficial in medical diagnostics, where the precise detection of low concentrations of biomarkers is essential for early disease diagnosis. Perovskite-based sensors have demonstrated exceptional quantitative performance metrics, significantly surpassing traditional materials in sensitivity and detection limits. For example, CH_3_NH_3_PbI_3_ perovskite photodetectors have achieved detection limits as low as 6.5 × 10^−12^ W/cm^2^ for near-infrared light, making them ideal for applications requiring high sensitivity [41]. Similarly, perovskite-based X-ray detectors have reported sensitivities exceeding 80 μC/Gy·cm^2^, which is several times higher than conventional silicon detectors. In biosensing, tin-based perovskite photodetectors have been shown to detect biomarkers at concentrations as low as 10^−12^ M, enabling early and precise disease diagnostics. These metrics underscore the suitability of perovskite materials for next-generation sensor applications, particularly in scenarios requiring ultra-low detection thresholds and high precision. The effectiveness of perovskite photodetectors in identifying faint light signals significantly enhances their utility in applications such as fluorescence imaging and various biosensing approaches [42,43]. By enabling the identification of biomarkers at greatly reduced concentrations, perovskite sensors make substantial contributions to advancing personalized medicine, ultimately improving patient outcomes [44].

### 3.3. Stability and Scalability Challenges

Despite their outstanding optical and electrical properties, perovskite materials face significant challenges associated with environmental stability and scalability of production methods for mass applications [45]. Notably, these materials can degrade under atmospheric conditions when exposed to moisture and light, which poses risks to their functionality and longevity [46]. In response to these challenges, advancements in material engineering have led to the development of hybrid perovskites that showcase enhanced stability [47]. Various strategies have been explored to improve the stability of perovskites in physiological environments, addressing their vulnerability to moisture, light, and temperature. Polymer encapsulation, such as with PMMA or PEO, effectively shields perovskites from environmental stress while preserving their optical and electronic properties. Surface passivation using materials like aluminum oxide or organic ligands has been shown to reduce defect sites and prevent degradation caused by interaction with biological fluids. Additionally, alloying with lead-free components, such as tin- or cesium-based perovskites, not only mitigates toxicity concerns but also enhances structural stability. For instance, tin-based perovskite sensors have demonstrated improved resistance to environmental conditions while maintaining high sensitivity. Combining these approaches with advanced fabrication techniques, such as roll-to-roll processing, could further enhance the practical applicability of perovskite-based devices in clinical settings [48]. Table 1 provides a clear summary of the discussed materials and their applications.

## 4. Plasmonic Nanomaterials in Optical Biosensing

Plasmonic nanomaterials (P-NM) are increasingly attracting attention due to their exceptional properties, including surface-enhanced Raman scattering (SERS), localized surface plasmon resonance (LSPR), plasmonic resonance energy transfer (PRET), and magneto-optical (MO) effects [65,66]. A variety of nanomaterials have been developed in this field, such as metal nanoparticles (MNP), bimetallic nanoparticles (bMNP), MNP-decorated carbon nanotubes (MNP-CNT), and MNP-modified graphene (MNP-GRP). The unique characteristics of these P-NMs position them as promising candidates for integration into optical biosensing systems. The optical property changes in plasmonic nanomaterials can be monitored based on the interactions between probe biomolecules and target viruses. Following bioconjugation, alterations in several optical properties such as fluorescence, plasmonic absorbance, and diffraction angle can be observed, enabling the detection of target biomolecules [67].

### 4.1. Plasmonic Phenomena and Applications

Plasmonic nanomaterials consist of sharp edges and uniform nanostructural arrangements that significantly enhance local electric fields. Gold and silver nanoparticles are particularly noteworthy for their ability to exhibit localized surface plasmon resonance (LSPR), which generates enhanced local electromagnetic fields that can greatly amplify the sensitivity of optical biosensors [68]. Achieving strong resonance depends on several factors, including the nanoparticle size, shape, composition, and the surrounding dielectric environment. For instance, smaller nanoparticles (<50 nm) typically exhibit sharper LSPR peaks, while anisotropic shapes, such as nanorods or nanostars, allow for tunable resonance wavelengths [69]. The dielectric constant of the surrounding medium plays a critical role, as a higher refractive index enhances resonance intensity. Additionally, material composition influences resonance; gold is preferred for its stability, while silver offers stronger resonance but suffers from oxidation. Optimizing these parameters is essential for designing plasmonic systems with maximal sensitivity for biosensing applications [2]. Consequently, plasmonic materials are increasingly employed in label-free biosensors capable of detecting biomarkers and single molecules with high specificity, thus eliminating the need for fluorescent or radioactive labeling [70,71]. For instance, LSPR-based systems utilizing non-spherical gold nanoparticles have been effectively used for the detection of viral antigens. One notable application involves an LSPR system developed for the detection of influenza virus, achieving a limit of detection (LOD) of 0.03 pg/mL in deionized water and 0.4 pg/mL in serum [44]. Furthermore, when QDs are positioned near P-NMs, the fluorescence intensity is enhanced through the PRET effect, facilitating sensitive monitoring of target viruses [50]. Similar techniques have been successfully employed for detecting Zika virus RNA, attaining a detection limit of 2.4 copies/mL. Additionally, studies demonstrated that iron oxide–gold (IO-Au) core/shell nanoparticle SERS platforms can monitor tuberculosis (TB) antigens with a LOD of 0.0511 pg/mL [72]. The alignment of nanoparticles through a magnetic field allows for simplified fabrication processes.

**Figure 3:**
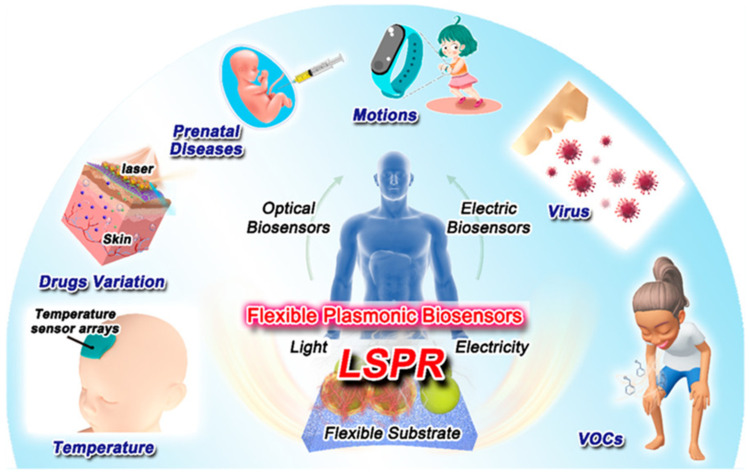
Application of plasmonic biosensors for biomarker detection in human samples. Illustrates their high sensitivity and rapid diagnostic capabilities, enabling early disease detection and monitoring of molecular changes in real time. Such advancements are critical for precision medicine and improving clinical outcomes. Reproduced from ref. [76] with permission from ACS Copyright 2021.

Moreover, a silver nanoparticle–graphene (AgNP-GRP) SERS platform showcased the capability of detecting methylated DNA, achieving an impressive LOD of 1.8 pg/mL while retaining the ability to be reused after cleaning [73]. Likewise, utilizing a similar strategy with gold–carbon nanotubes (Au-CNT), the influenza virus was detected with a sensitivity of 3.4 plaque-forming units per milliliter (PFU/mL), outperforming traditional detection methods [74]. The development of sophisticated flexible plasmonic biosensors is crucial for rapid and accurate medical diagnoses. For example, optical biosensors incorporating plasmonic and photonic crystal bandgap structures demonstrated maximum sensitivities and figures of merit (FOM) values of 718.6 nm/RIU, 714.3 nm/RIU, and 156.217 RIU−1, 60.1 RIU−1, respectively, specifically for the detection of basal cell carcinoma [75]. These biosensors enable the monitoring of various physiological indicators, including ions, blood glucose levels, and body temperature Figure 3. This capability streamlines experimental processes and minimizes potential interferences, contributing to the overall reliability of diagnostic assays.

### 4.2. Innovations in Biosensing

**Figure 4:**
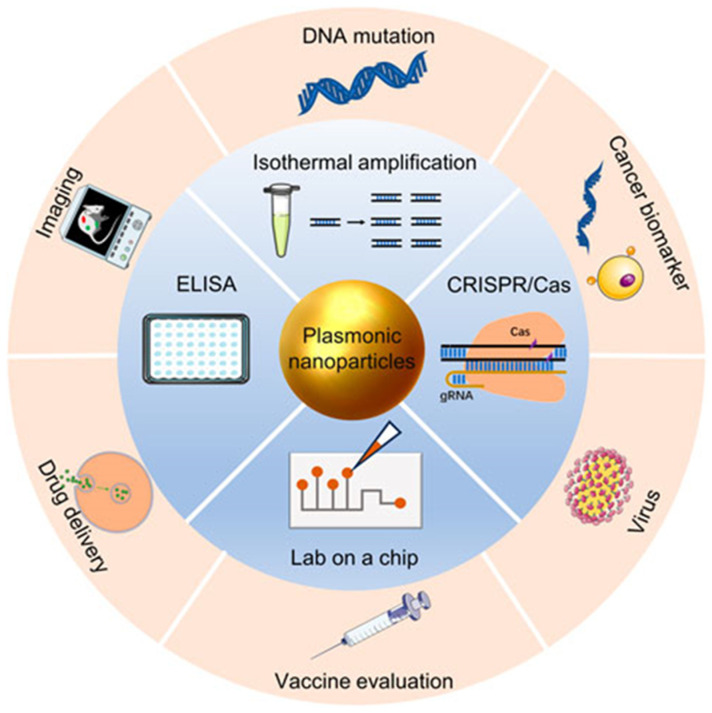
Overview of current challenges and emerging solutions in nanoplasmonic biosensors. It details innovations that address material stability, biocompatibility, and enhanced sensitivity, which are critical for effective integration into clinical diagnostics and for improving real-time disease detection. Reproduced from ref. [82].

Recent advancements in plasmonic nanomaterials, particularly gold and silver nanoparticles, have significantly improved biomarker detection and diagnostics. Achieving strong plasmonic resonance requires precise control over fabrication parameters such as particle size and aspect ratio, as well as post-synthesis modifications to stabilize the plasmonic response. For example, increasing the aspect ratio of nanorods shifts the resonance wavelength into the near-infrared region, which is particularly useful for deeper tissue imaging [77]. The surrounding medium’s refractive index and the proximity of particles in a colloidal solution can also affect resonance strength, highlighting the need for controlled aggregation or surface functionalization strategies [78]. These design considerations ensure enhanced sensitivity and specificity in plasmonic biosensors. Their strong localized surface plasmon resonance (LSPR) properties enable the identification of subtle refractive index changes, which are critical for the early detection of diseases such as cancer, cardiovascular issues, and neurological disorders. Moreover, the enhanced capabilities of these nanomaterials markedly improve diagnostic methods, particularly for neurological disorders, ultimately aiming to enhance patient outcomes through timely and precise diagnoses [79]. These biosensors facilitate rapid real-time analysis, making them especially suitable for point-of-care diagnostics, where prompt results can significantly influence clinical decision-making. The advancements in plasmonics have also paved the way for the development of nanomaterials capable of effectively controlling the emission of molecular or nanosized photoluminescent materials through recent innovations in photonic crystal microcavities [80]. This plasmonic technology simplifies label-free detection methods while enabling the simultaneous identification of multiple targets within a single assay. Furthermore, it provides high-resolution imaging and comprehensive molecular data, which are vital for improved disease characterization and monitoring; specific ligands or antibodies enhance targeting capabilities, allowing for precise diagnostics tailored to the individual needs of each patient [81]. The progress in these advanced sensing technologies underscores the potential of plasmonic nanomaterials to revolutionize medical diagnostics Figure 4.

Despite significant progress, ongoing challenges persist in the realm of plasmonic biosensing, particularly regarding the durability of materials and devices in complex biological environments. Achieving high sensitivity and specificity in such settings is paramount for effective clinical applications. Future research may focus on the integration of plasmonic biosensors with microfluidic systems, allowing for the miniaturization and automation of diagnostic assays. However, designing plasmonic nanomaterials to achieve strong resonance requires challenges to be addressed such as reproducibility in synthesis, stability under biological conditions, and compatibility with microfluidic platforms. Strategies like alloying gold and silver or coating with stabilizing layers (silica or polymers) can mitigate degradation and maintain resonance properties. Moreover, computational modeling of plasmonic structures can aid in predicting and optimizing resonance conditions for specific applications, ensuring that these sensors achieve high performance in practical diagnostic environments [83]. The versatility of gold and silver nanoparticles facilitates their use across a wide array of applications. This integration could transform diagnostics, offering compact, efficient, and highly sensitive testing options that potentially increase accessibility to advanced healthcare solutions.

## 5. Organic Semiconductors in Bio-Optoelectronics

Organic semiconductors are organic materials characterized by distinct electronic properties that lie between those of traditional conductors and insulators. Specifically, the band gap between the valence and conduction bands of organic semiconductors is relatively lower than that found in insulators yet higher than that seen in conventional conducting polymers [51]. These semiconductors are primarily composed of carbon-based molecules or polymers, often referred to as π-conjugated systems, which facilitate the movement of charge carriers (electrons and holes) through their conjugated molecular structures [84].

### 5.1. Properties of Organic Semiconductors

**Figure 5:**
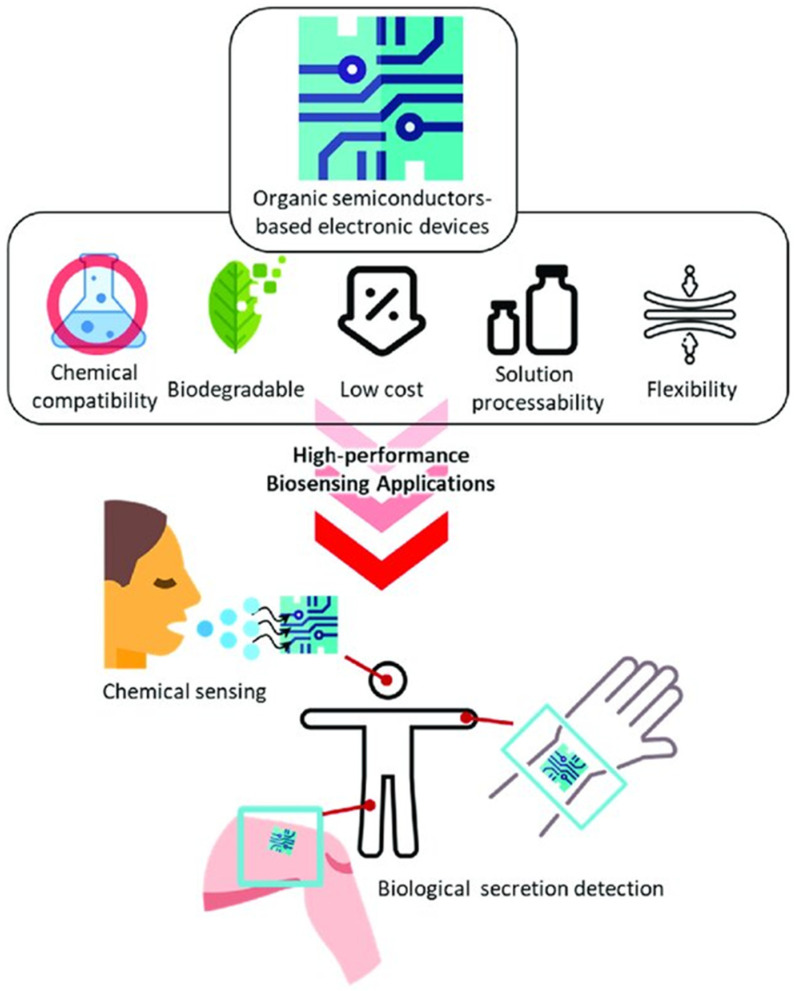
Utilization of organic semiconductors in biomedical imaging and therapeutics. A schematic representation of their biocompatibility and functional flexibility for non-invasive diagnostics and targeted treatment options. Reproduced from ref. [89].

Organic semiconductors possess several advantageous properties that enhance their suitability for bio-optoelectronic applications, including a low dielectric constant (ε) that influences their electronic behavior, mechanical flexibility that facilitates integration with diverse substrates, including flexible ones, and tunable electronic characteristics that allow for the modification of electronic properties through chemical alterations, thereby adapting to various applications [85,86]. Organic semiconductors can be processed at relatively low temperatures, facilitating the formation of well-defined films and layered structures without the need for high-temperature techniques. Additionally, they have elevated triplet-state energy levels and critical transport characteristics [87]. Such attributes allow organic conducting polymers to be utilized in various electronic devices, exhibiting a well-defined energy band gap that allows them to transition between conductive and insulating states. Prominent examples of these organic polymeric semiconductors include polyparaphenylenevinylene (PPV), polyparaphenylene (PPP), polyfluorene (PF), and polyfluorene copolymers [88]. Both organic semiconductors offer significant advantages, such as solution processability and low-temperature deposition, making them suitable for diverse electronic and optoelectronic applications Figure 5.

The intrinsic flexibility of organic semiconductors is particularly advantageous for conforming to biological tissues, thereby facilitating the development of medical devices that adhere comfortably to the body’s anatomical contours without causing discomfort [90]. These materials are increasingly recognized for their potential in a wide array of bio-optoelectronic devices, such as organic photovoltaic cells (OPVs), which serve to power implantable devices including pacemakers and neurostimulators [91]; organic light-emitting diodes (OLEDs), which are employed in biomedical imaging applications, notably in optogenetics and photodynamic therapy [92]; and organic photodetectors that play a critical role in both in vitro and in vivo biosensing applications, enabling the detection of biomarkers to monitor physiological parameters [52]. Moreover, the application of organic semiconductors extends to organic field-effect transistors (OFETs), in which these materials act as active channel components, allowing the fabrication of flexible and low-power transistor devices [93]. Additionally, organic sensors exploit the heightened sensitivity of organic semiconductors to monitor variations in environmental parameters, including gas concentrations and biomolecular interactions. Ongoing research efforts are focused on advanced material engineering, innovative doping techniques, and the development of novel device architectures, all aimed at enhancing the performance and stability of organic semiconductors while systematically addressing the existing limitations within the field [94]. Such advancements hold significant promise for the integration of organic semiconductor technology in next-generation bio-optoelectronic applications.

### 5.2. Advances in Organic Photodetectors

Recent advancements in organic photodetectors have garnered significant attention due to their role as key technologies in advanced imaging systems and sensors [95]. These devices are characterized by their lightweight, ultra-flexible, environmentally friendly, and cost-effective nature, alongside exhibiting long-term stability. They enable real-time monitoring, produce high-quality images, enhance response speed, and minimize dark noise current, offering considerable advantages over traditional inorganic materials. Such attributes have catalyzed innovative applications within the health monitoring sector [96,97]. Organic photodetectors have been successfully utilized to monitor critical physiological parameters, including heart rate and respiration rate, through dynamic signal acquisition techniques. These detectors leverage the optical and electrical properties of organic materials to capture blood flow patterns (heart rate) and thoracic movements (respiration rate), as highlighted in other studies [98]. Such advancements have expanded their utility in wearable health monitoring systems. Their applications extend to digital cameras, medical imaging, light-based communication systems, and optical sensors used for environmental monitoring and industrial purposes.

Moreover, the flexibility and lightweight attributes of organic materials facilitate the development of wearable and conformable photodetectors designed for health monitoring, biometric sensing, and smart textile applications [99,100]. They are particularly well-suited for such purposes due to their low skin-contact impedance of 15 kΩ·cm^2^ at 100 Hz, enabling high-quality detection of electrophysiological signals [101]. Additionally, skin-integrated multimodal systems developed from these materials can accurately and continuously measure vital signs, including heart rate, respiration rate, and blood pressure, in a cuff-less manner while also monitoring arterial oxygen saturation, even under dynamic conditions. Importantly, organic photodetectors exhibit strong anti-interference capabilities [102], providing economic and non-invasive diagnostic options that enhance patient comfort and compliance. For example, saliva serves as a valuable source of biomarkers, including interleukins (IL-1β and IL-8) and matrix metalloproteinases (MMP-8 and MMP-9), which are linked to a range of diseases such as cancer, arthritis, cardiovascular diseases, and inflammatory disorders [103]. Integrating these diagnostic devices into wearable formats significantly enhances their potential for real-time health monitoring and expands their practical applications.

### 5.3. Challenges and Innovations

Despite the promising applications of organic semiconductors, one primary challenge remains: their stability in physiological environments, which can significantly impact long-term device performance. In bio-optoelectronic applications, degradation due to humidity, temperature fluctuations, and exposure to biological fluids poses risks to the long-term functionality of these materials. One substantial hurdle involves the complexity of processing organic materials, as well as their inadequate mechanical softness, particularly when compared to traditional inorganic elements like silicon (Si) and germanium (Ge). Additionally, there is a critical need to reduce toxicity in organic materials to enhance the biocompatibility of organic photodetectors (OPDs) [104]. Consequently, current research efforts are focused on developing more stable organic materials alongside innovative encapsulation methods that safeguard these devices against environmental degradation. Recent advancements in nanoparticle-incorporated elastomeric composites, which feature modified surface morphology, have demonstrated promise in protecting biodegradable electronic devices against environmental degradation. Examples of materials used include poly(l-lactide-co-ε-caprolactone) (PLCL), polytetrafluoroethylene (PTFE), and silicon dioxide (SiO_2_) nanoparticles [105,106,107]. The long-term durability of organic semiconductor devices in medical applications relies on these innovations, thus paving the way for broader implementation of these devices in healthcare settings.

Organic and inorganic semiconductors each have distinct advantages and limitations in diagnostic applications. Organic semiconductors, such as π-conjugated polymers, offer mechanical flexibility, lightweight properties, and solution processability, making them ideal for wearable and implantable devices [108]. However, they are more prone to degradation in physiological conditions due to their susceptibility to oxidation, humidity, and UV exposure. Conversely, inorganic semiconductors, such as silicon and perovskites, exhibit superior electronic properties, photostability, and higher carrier mobilities but lack the mechanical flexibility required for certain applications. To address the stability challenges of organic semiconductors in physiological environments, encapsulation techniques have been widely adopted. Thin layers of materials such as polyethylene oxide (PEO), polyvinyl alcohol (PVA), or siloxane-based polymers can effectively shield organic semiconductors from moisture and oxygen [109]. For instance, encapsulating organic photodetectors with ultra-thin atomic layer deposition (ALD) coatings has significantly improved their operational lifetime in biological conditions. Similarly, for inorganic semiconductors, hybrid encapsulation strategies combining polymer matrices with inorganic barriers like aluminum oxide or silicon nitride have demonstrated enhanced resistance to environmental stress [110]. By integrating these encapsulation approaches, the operational stability and durability of both organic and inorganic semiconductors in diagnostic devices can be markedly improved, extending their applicability across diverse medical scenarios.

## 6. Integration of Functional Materials in Advanced Imaging Technologies

Functional materials play a crucial role in advancing imaging technologies by enhancing contrast, resolution, and depth penetration. Low-dimensional van der Waals materials, such as TMDs and graphene derivatives, have recently gained attention for their unique optoelectronic properties, which enable advanced functionalities in medical imaging and diagnostics [111]. These materials facilitate the precise visualization of biological structures through their strong optical absorption and photoluminescence, particularly in near-infrared regimes, enabling deeper tissue penetration and high-resolution imaging [5]. These improvements facilitate the precise visualization of biological structures and tissues in both clinical and research settings. For example, recent innovations have enabled the integration of functional materials into devices for heart rate and respiration rate monitoring, utilizing photonic mechanisms to detect subtle changes in blood flow and thoracic movements. Technologies such as wearable PPG sensors [112] have proven highly effective in real-time health monitoring applications. The application of functional materials in these technologies holds significant promise for improving diagnostic accuracy, monitoring disease progression, and developing individualized treatment plans. By improving sensitivity and specificity across various imaging modalities, functional materials enhance the overall performance of diagnostic systems. For instance, 18-Fluoro-deoxyglucose positron emission tomography (^18^F-FDG PET/CT) has limited diagnostic accuracy in detecting lobular breast cancer bone metastases; however, emerging functional imaging approaches are enabling improved molecular imaging that can more accurately identify specific breast cancer subtypes [113]. Additionally, computer-aided diagnostics (CAD) in mammography integrates anatomical and functional breast imaging, thereby refining cancer detection. Recent advancements in deep learning have enabled CAD systems to identify up to 90% of all cancers, although challenges related to false positives remain an ongoing target for improvement [114]. Enhanced imaging contrast, resolution, and depth penetration are essential for accurately visualizing biological tissues and structures. For example, nanoparticles in Optical Coherence Tomography (OCT) can increase image contrast by better-delineating tissue structures, ultimately improving real-time diagnostic capabilities.

Metal oxide nanoparticles, including magnesium oxide (MgO), zinc oxide (ZnO), nickel oxide (NiO), and titanium dioxide (TiO_2_), have been shown to enhance film stability during light exposure, which in turn improves imaging clarity and reliability [115]. Furthermore, luminescent hollow spherical nanoparticles enhance imaging contrast by interacting with tissues at specific wavelengths, facilitating precise visualization of structural details [116]. Photoacoustic imaging also significantly benefits from the incorporation of functional materials that enhance signal intensity and spatial resolution. This technique, which relies on the photoacoustic effect to detect tissue abnormalities, has become increasingly effective through the use of semiconducting polymer nanoparticles, which provide high photostability, biocompatibility, and tunable absorption properties due to their chemically modifiable structures [117,118]. Recent innovations, such as ultrasonic transducer arc arrays, enable rapid, high-resolution photoacoustic tomography (PAT), allowing for a complete 360° scan in just 15 s, significantly accelerating the imaging process while enhancing clinical applicability.

### 6.1. Enhancing Imaging Modalities

Advancements in imaging modalities through the use of functional materials have led to more accurate and detailed tissue characterization, which is vital for early disease diagnosis and treatment planning. By enhancing contrast and spatial resolution, these materials contribute to a clearer delineation of tissue architecture [119]. In OCT, the use of functional nanoparticles improves image contrast, thereby enabling precise tissue visualization and real-time diagnostic applications. Furthermore, functional materials are critical in CAD systems for mammography, where the integration of anatomical and functional imaging has improved breast cancer detection, particularly when combined with deep learning techniques [114]. Metal oxide nanoparticles such as MgO, ZnO, NiO, and TiO_2_ enhance the stability of imaging films during light exposure, which is crucial for high-resolution imaging applications. These nanoparticles provide enhanced photostability and reliability, sustaining image quality during prolonged exposure [115]. Luminescent hollow spherical nanoparticles also contribute to improved imaging contrast, allowing for detailed visualization of tissue boundaries and better differentiation between pathological structures [116]. In photoacoustic imaging, the use of functional materials significantly enhances signal strength and resolution, facilitating the detection and characterization of tissue abnormalities. Table 2 summarizes the advancements in imaging technologies achieved through functional materials.

### 6.2. Development of Novel Contrast Agents

**Figure 6:**
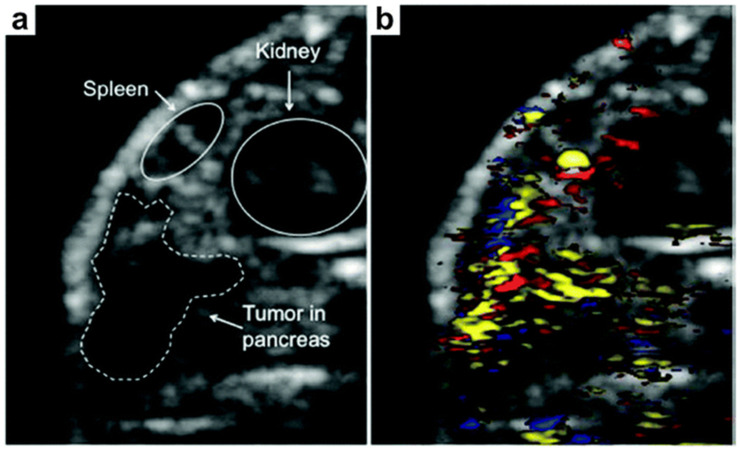
Dual-modality imaging of pancreatic tumors using targeted nanoplates. (**a**) Ultrasound Imaging of Pancreatic Tumor: Displays an ultrasound image of a pancreatic tumor in a mouse model, demonstrating non-invasive tumor localization and monitoring potential. (**b**) Photoacoustic Imaging with Targeted Silver Nanoplates: Shows a photoacoustic image of the tumor using silver nanoplates conjugated shows (yellow). This modality differentiates oxygenated (red) and deoxygenated (blue) blood within the tumor, providing detailed vascular mapping and enhanced tumor visualization for precise diagnostic assessments. Reproduced from ref. [132] with permission from ACS Copyright 2012.

The design and development of novel contrast agents based on functional materials have transformed imaging techniques by enabling targeted contrast enhancement. This includes advances in 3D bioprinting, engineered nanoparticles, and Photon Counting Computed Tomography (PCCT) technologies, which create traceable, custom-designed scaffolds in precision medicine. Materials such as AuMA (Gold Mercaptoalkylamide) and Gd_2_O_3_ (Gadolinium oxide) nanoparticles have been utilized for non-invasive material discrimination and quantification of contrast agents [130]. Engineered fluorophores and nanoparticles that selectively bind to specific tissues or biomarkers contribute to more accurate imaging, which is essential for early cancer diagnosis and treatment monitoring. Semiconducting polymer nanoparticles are notable for their high photostability, biocompatibility, and chemical tunability, making them ideal candidates as photoacoustic contrast agents [117,118]. These materials facilitate a higher quantum yield, thereby improving signal intensity and spatial resolution. Advanced ultrasonic transducer arrays in photoacoustic tomography, for example, can perform comprehensive 360° scans in just 15 s, resulting in quick and high-resolution tissue imaging. Additionally, multipurpose magnetic nanoplatforms have emerged as key components for multimodal imaging probes, enhancing image contrast and facilitating early disease diagnosis and treatment by providing detailed molecular-level information [120,131]. Tailored fluorophores, specifically designed to target malignant tissues while distinguishing them from healthy tissues, further enhance the diagnostic accuracy of these contrast agents, supporting more precise therapeutic interventions Figure 6.

### 6.3. Future Directions: Functional Materials and Artificial Intelligence (AI) in Precision Imaging

The integration of functional materials with advanced machine learning algorithms signifies a major trend in medical imaging, leading to the development of smart systems capable of automated diagnostics. It is anticipated that these evolved imaging systems will enhance personalized diagnostic methods, aligning them more closely with individual patient anatomy and physiology. For example, future research on degenerative diseases may focus on sleep-related disorders, as sleep disturbances are common among patients with these conditions; imaging could provide objective, quantitative insights into these issues [6]. Laser Speckle Contrast Imaging (LSCI), a high-resolution, non-invasive technique for assessing microcirculatory blood flow, is currently undergoing enhancements to its quantitative analytical capabilities. Future advancements in endoscopic technology and the integration of artificial intelligence with LSCI could enhance its diagnostic value by facilitating its combination with other imaging modalities for comprehensive patient assessments [133]. This integration will create more adaptive imaging systems capable of responding to specific diagnostic needs in real time, ultimately improving diagnostic accuracy and patient care outcomes. The synergy between functional materials and AI technologies will continue to increase the efficiency and adaptability of imaging modalities, leading to significant advancements in disease diagnosis and management [134]. Consequently, the future of medical imaging is poised for transformative changes through the continued integration of functional materials and AI, enabling precision diagnostics, automated imaging, and personalized care. These developments have the potential to revolutionize the landscape of disease diagnosis and treatment, equipping clinicians with more robust and accurate tools for managing a wide range of medical conditions.

Specific case studies illustrate how AI-driven imaging systems, combined with functional materials, have significantly improved diagnostic outcomes. For example, an AI-assisted photoacoustic imaging system using semiconducting polymer nanoparticles enabled the early detection of breast cancer with over 95% accuracy, demonstrating enhanced sensitivity in identifying vascular anomalies associated with tumor growth [135]. Similarly, the application of AI algorithms in analyzing quantum dot-based fluorescence imaging has accelerated the detection of biomarkers in neurological disorders, reducing the diagnostic time by nearly 50% compared to conventional methods [136]. These examples highlight the synergistic role of AI and functional materials in achieving higher diagnostic precision and efficiency. Functional materials also play a pivotal role in improving the cost-effectiveness of imaging technologies. QDs, for instance, offer high fluorescence stability and multiplexing capabilities, reducing the need for multiple imaging agents and repeated scans. Similarly, organic semiconductors enable the fabrication of lightweight and flexible imaging devices at a lower cost through roll-to-roll manufacturing processes. Perovskite-based detectors, with their high sensitivity and low material cost, have shown the potential to replace expensive silicon-based systems in X-ray imaging. By integrating these materials into diagnostic platforms, it becomes possible to reduce production and operational costs, making advanced imaging technologies more accessible for widespread clinical use.

## 7. Challenges and Opportunities

Integrating advanced functional materials into medical imaging and diagnostic devices offers significant opportunities to enhance precision, sensitivity, and therapeutic potential. However, realizing these benefits necessitates overcoming various challenges, particularly those related to scalability, sustainability, and economic feasibility. These challenges are essential for advancing functional materials such as QDs, perovskites, and van der Waals materials for commercial applications. Complex synthesis processes often hinder scalability, but transitioning from batch synthesis to continuous flow systems can improve reproducibility and quality. Sustainability concerns arise from toxic precursors and energy-intensive production methods, as seen with cadmium-based QDs, prompting research into greener methodologies that utilize alternative materials, such as InP QDs, and incorporate plant-based reducing agents. Economic feasibility remains crucial; strategies such as utilizing low-cost raw materials and scalable manufacturing techniques, including roll-to-roll processing and self-assembly, are vital for reducing costs. Targeted research and collaboration among diverse scientific disciplines are essential to developing economically viable and environmentally sustainable materials for practical applications in medical diagnostics. This section highlights the challenges and opportunities while emphasizing the pivotal role of interdisciplinary collaboration as a key enabler in overcoming these obstacles.

### 7.1. Material Compatibility and Biocompatibility

The integration of functional materials within medical devices presents substantial challenges, especially regarding material compatibility and biocompatibility in biological environments. Biocompatibility, or the ability of materials to function without adverse physiological responses, is crucial for ensuring patient safety, especially in implants. Surface treatments such as polishing, etching, and coating can significantly reduce infection risks, thereby enhancing biocompatibility [137]. Nevertheless, foreign body reactions (FBR) remain a concern as the immune system may respond negatively to these materials, causing complications such as inflammation, infection, or tissue damage [138]. Long-term stability in dynamic biological environments is another pivotal aspect, as variations in pH, temperature, and exposure to biological fluids can undermine the structural integrity and functionality of materials. Surface modifications, including functional group attachment and coatings, enhance stability and targeting in vivo [139]. Addressing compatibility and stability issues is essential for successfully translating these advanced materials from laboratory research to clinical usage. In the case of low-dimensional van der Waals materials, challenges include scalability, ambient stability, and integration into existing device architectures. Recent advancements in passivation techniques and heterostructure engineering; however, have begun to show promise in overcoming these limitations, paving the way for their adoption in clinical diagnostics [140,141]. Continued interdisciplinary collaboration is necessary to realize the full potential of these materials in optoelectronic medical technologies.

### 7.2. Regulatory and Ethical Considerations

As functional materials in medical technologies progress toward clinical application, they confront various regulatory and ethical challenges focused on patient safety. Regulatory bodies, such as the FDA, play a critical role in ensuring the safety and efficacy of devices that incorporate nanomaterial components. However, the economic feasibility of scaling up production must align with these regulatory requirements. For instance, ensuring batch-to-batch consistency in scaled-up production can significantly increase costs due to stringent quality control measures. Developing automated, cost-effective quality assurance tools—such as in-line spectroscopy or AI-driven defect detection systems—can mitigate these challenges, ensuring compliance without compromising economic viability. The approval processes for these devices mandate extensive testing, including clinical trials and post-market surveillance to monitor adverse effects and facilitate necessary updates [142,143]. Furthermore, the inclusion of functional materials in medical diagnostics raises ethical concerns regarding equitable access, as these advanced technologies may be prohibitively expensive, potentially creating disparities in healthcare access [144]. The growing integration of digital health technologies complicates this landscape, as significant amounts of personal health data are collected, raising concerns about data security and patient privacy. Ethical considerations encompass issues such as patient consent, transparency in algorithmic decision-making, and addressing biases inherent in AI and machine learning systems, especially in diagnostic contexts [2]. Understanding and addressing these regulatory and ethical frameworks is vital for fostering public trust and supporting the seamless integration of new diagnostic technologies into healthcare practices.

### 7.3. Opportunities for Collaboration

Addressing the multifaceted challenges associated with advanced functional materials in medical imaging and diagnostics requires robust interdisciplinary collaboration across materials science, bioengineering, clinical medicine, and regulatory science [145]. For example, materials scientists can collaborate with bioengineers to create materials that satisfy stringent biocompatibility requirements while providing stability in biological environments [146]. Such collaborative partnerships are critical in developing diagnostic technologies that integrate medical imaging, AI, and machine learning to enhance disease detection accuracy and refine clinical decision-making processes [147]. Technological advancements further facilitate collaborations across diverse healthcare domains. For instance, the integration of AI in digital radiology, pathology, and dermatology enhances communication between clinics, hospitals, and patient monitoring and intervention centers (PMICs), streamlining expert consultations and optimizing workflows to improve diagnostic precision [148]. Additionally, multifunctional nanoplatforms such as the Fe_3_O_4_@SiO_2_@NaYF_4_^3+^ nano-platform, designed for biocompatibility and high-resolution imaging capabilities, support dual-mode in vivo imaging and magnetic-driven applications, offering promising avenues for deep tissue analysis [149]. This innovative platform, characterized by its unique core/shell structure and exceptional photostability, enables accurate, high-resolution imaging, thus positioning it as a valuable tool for complex diagnostic procedures. Collaboration with clinical practitioners is equally essential for gathering practical insights regarding the usability and effectiveness of these diagnostic tools in real-world settings. Concurrently, regulatory scientists can guide the technology development process, ensuring compliance with safety standards, a critical factor for bridging scientific innovation and clinical applications [150]. This collaborative approach is vital for accelerating advancements in medical diagnostics and ensuring that these pioneering technologies effectively reach and benefit patients, ultimately enhancing the quality and accessibility of healthcare outcomes.

## 8. Conclusions and Future Perspectives

Functional materials exhibit transformative potential in the field of medical diagnostics by facilitating the development of photonic and optoelectronic devices that enhance imaging and sensing capabilities. These advancements are crucial for early disease detection and personalized medicine, promising improved health outcomes for patients. Despite considerable progress, challenges related to material biocompatibility, stability, scalability, and compliance with regulatory standards persist, underscoring the need for sustained research efforts and interdisciplinary collaboration. Future efforts should focus on several specific research areas, including the exploration of biocompatible and sustainable materials, which is essential for developing next-generation materials with enhanced biocompatibility and lower environmental impact. Heavy metal-free quantum dots, such as indium phosphide-based systems, and carbon-based nanomaterials represent promising candidates. Additionally, the adoption of green synthesis techniques utilizing low-energy processes and plant-based reducing agents may significantly reduce the ecological footprint associated with material production.

Advancements in surface engineering, particularly in surface passivation, encapsulation, and hybrid material systems, can also improve the stability and durability of functional materials under physiological conditions. Effective strategies, such as polymer matrix encapsulation for perovskites and silica coatings for plasmonic nanomaterials, have shown the potential to enhance clinical viability. The integration of artificial intelligence (AI) and machine learning techniques can optimize material properties, device design, and diagnostic algorithms, accelerating the development of high-performance diagnostic tools. AI-driven imaging systems that utilize functional materials like quantum dots have already demonstrated superior diagnostic accuracy and efficiency, heralding broader applications across various medical contexts. Furthermore, cost-effective manufacturing techniques need to be prioritized, with collaborative efforts between academia and industry aimed at scaling up manufacturing processes. Innovations such as roll-to-roll nanolithography and continuous flow production systems are critical for reducing production costs while maintaining the high-quality standards essential for clinical applications. The development of multifunctional devices that combine imaging and therapeutic capabilities, exemplified by theranostics devices employing plasmonic nanomaterials or quantum dots, presents significant prospects for integrated disease management.

Interdisciplinary collaboration remains vital in driving advancements in functional materials for medical diagnostics. Partnerships among researchers in materials science, bioengineering, computer science, and clinical medicine will facilitate joint initiatives, such as the creation of wearable health monitoring devices that integrate organic semiconductors and AI algorithms, potentially revolutionizing real-time health monitoring. The future of functional materials in medical diagnostics hinges on fostering an environment of innovation and collaboration across diverse scientific disciplines. By addressing existing challenges and delving into these targeted research areas, the full potential of functional materials can be unlocked, paving the way for the development of next-generation diagnostic tools that are efficient, cost-effective, and environmentally sustainable. These efforts promise not only to transform diagnostic technologies but also to promote more equitable and accessible healthcare solutions globally.

## Figures and Tables

**Table 1:** Key functional materials in photonic and optoelectronic devices for medical diagnostics.

S. No	Functional Material	Key Properties	Diagnostic Applications	Challenges	Sensitivity/Performance Metrics	Ref.
1.	Quantum Dots (QDs)	The material exhibits photostability, high quantum yield, and size-tunable fluorescence.	Treating infectious diseases and cancer; high-resolution imaging and real-time biosensing.	Challenges with low stability in physiological conditions; potential toxicity, particularly with cadmium-based QDs.	Detection limits as low as 10^−12^ M for biomarker sensing.	[17,23]
2.	Perovskites	The device features long charge carrier diffusion lengths and high light-absorption coefficients.	The material is widely utilized in X-ray detectors and photodetectors for medical imaging.	Extremely sensitive to temperature, light, and moisture; scalability and long-term stability issues.	X-ray sensitivity exceeding 80 μC/Gy·cm^2^.	[37,38]
3	Plasmonic Nanomaterials	Shows magneto-optical effects, surface-enhanced Raman scattering (SERS), and localized surface plasmon resonance (LSPR).	Widely utilized in pathogen and biomarker detection, including cancer and viral diagnostics.	Reproducibility and stability in complex biological environments.	Detection limits down to 2.4 copies/mL for viral RNA.	[49,50]
4	Organic Semiconductors	Flexibility, and lightweight characteristics.	Utilized in organic photovoltaic cells, for wearable and implantable sensors for continuous patient monitoring.	Common physiological scenarios include moisture degradation, environmental instability, and shortened lifespan.	Stability enhancement with encapsulation; operational lifetimes extended by >30%.	[51,52]
5	Aptamer-Embedded Nanomaterials	Highly stable, tunable, and small in size.	Deep cellular analysis, imaging, gene editing, drug delivery, and cancer diagnosis.	Susceptible to nuclease degradation; challenges in reliable synthesis.	Sensitivity up to 95% in cancer biomarker detection.	[53]
6	MXene-Based Materials	High biocompatibility and ion transport characteristics.	Next-generation structure for (bio)sensing, imaging, and cancer diagnostics.	High energy consumption; environmental concerns.	High signal-to-noise ratio in biosensing applications.	[54]
7	Polymers	Rapid and accurate diagnostic capabilities.	Implemented in biodecorated membranes and polymeric nanoparticles.	Bacterial infections and sterilization issues.	Detection efficiency > 90% in polymer-based biosensors.	[55]
8	Hexagonal Boron Nitride (hBN)	Nonlinear optics, and single-photon emission at room temperature.	Suitable for deep UV emitters, detectors, and surface-enhanced IR absorption microscopy.	Material processing and nano-prototyping challenges.	IR absorption enhancement by 25% compared to traditional materials.	[56]
9	Polyethylene Glycol (PEG) Hydrogels	Electrical conductivity and light transmission.	Used for phototherapeutic devices, LEDs, and photodiodes.	Chemical instabilities and inconsistencies.	Conductivity improvements by 20% in advanced hydrogels.	[57]
10	Organic Frameworks	Eco-friendly and cost-effective.	Utilized in electrochemical biosensors, and continuous monitoring devise.	Stability, repeatability, and selectivity issues.	Detection limits improved to 1.2 μM in biosensing applications.	[58,59]
11	Lignin nanoparticles	Electromagnetic shielding, and high thermal stability.	Broad absorption of radiation frequency and protect medical devices.	Environmental challenging.	Highly sensitive in selective binding and signal amplification mechanism.	[60,61]
12	Piezoelectric polymer	Strong electroactive responses and high dielectric constant.	Utilized in tissue-related high-quality two-dimensional ultrasound imaging.	Uniform electrical polarization across large areas.	Highly sensitive to environmental conditions, and values in the range of 20–30 pC/N for imaging.	[62]
13	Colloidal quantum dots.	Size-Tunable Optical Features and High spectral tunability.	Thermal imaging, biosensing and in vivo imaging.	Regulatory approval, stability and cost of production.	Photodetector to detect weak signals in the presence of noise.	[63]
14	GaN (Gallium Nitride)	Good crystallinity and operational stability.	Enhance the photodetection (UV fluoresce) and fast biological markers detection.	Optimizing device efficiency and compatibility with current methodologies.	Highly sensitive in convert light into electrical signals effectively.	[64]

**Table 2:** Advancements in imaging technologies through functional materials.

S. No.	Imaging Technology	Enhancing Functional Material	Diagnostic Benefit	Example Use Case	Sensitivity/Performance Metrics	Ref.
1	Optical Coherence Tomography (OCT)	Metal oxide nanoparticles	Increases image contrast, stability under light exposure	Real-time tissue visualization	Improved resolution by 20% compared to conventional materials	[115,116]
2	Photoacoustic imaging (PAI)	Semiconducting polymer nanoparticles	Possess enhanced signal intensity, biocompatibility	Detection of tissue abnormalities	Sensitivity to micrometer-level tissue changes	[117,120]
3	Fluorescence imaging	QDs, organic dyes	Useful for multiplexed biomarker imaging	Cancer diagnosis, monitoring	Detection limits as low as 10^−12^ M in biomarker sensing	[23,25]
4	Laser Speckle Contrast Imaging (LSCI)	Functionalized nanoparticles	Implemented in quantitative blood flow assessment	Neurodegenerative disease diagnostics	Blood flow resolution enhanced by 15%	[6,121]
5	High signal-to-noise ratio (SNR)	Second near-infrared (NIR-II)	Helps in deeper tissue penetration and high-resolution image development	Medicine and biological research.	Tissue penetration up to 3 cm with high resolution	[122]
6	Enhance light absorption and energy transfer processes	Semiconductors and plasmonic metals	Used in optical imaging and suggests future research directions	Medicine and biological research.	Light absorption efficiency increased by 30%	[123]
7	Magnetic Resonance Imaging (MRI)	Functional nanomaterials	It targets specific sites in vivo, visualizing specific tissues or abnormalities	Medical diagnosis and disease detection,	Enhanced signal intensity in target-specific imaging	[124]
8	Photoacoustic imaging (PAI) with computed tomography (CT)	Polymer-decorated transition-metal nanomaterials	It exhibits excellent photothermal conversion efficiency	Multimodal imaging diagnosis and cancer therapy	Conversion efficiency >90% for photothermal imaging	[125]
9	Strong near-infrared optical absorption.	QDs	Helpful in reducing toxicity, and contain fluorescent imaging capabilities	Excellent in vivo tumor imaging.	Tumor imaging sensitivity increased by 25%	[126]
10	Photoluminescence	Nanographene oxides	Implemented in biomedical imaging and stable photoluminescence	Visualize and track migratory cancer cells	Enhanced imaging contrast by 20%	[127]
11	Ultrasound imaging	Gas-filled microbubbles	Improved contrast for soft tissue imaging	Visualize real-time blood flow	Enhanced contrast to tissue by 40%	[128]
12	Raman imaging	Gold nanoparticles with Raman reporters	Enhances the Raman signal intensity for molecular imaging	Single-cell analysis	Amplification of the signal up to 10^6^-fold	[129]

## Data Availability

No data were used for the research described in the article.

## Abbreviations

^18^F-FDG PET/CT	18-Fluoro-deoxyglucose positron emission tomography
AgNP-GRP	Silver nanoparticle-Graphene
AI	Artificial Intelligence
Au-CNT	Gold Carbon Nanotubes
AuMA	Gold Mercaptoalkylamide
bMNP	Bimetallic Nanoparticles
CAD	Computer-Aided Diagnostics
CdSe@ZnS	Cadmium Selenide Zinc Sulfide
CNT	Carbon Nanotubes
DNA	Deoxyribose Nucleic Acid
FBR	Foreign Body Reactions
FDA	Food and Drug Administration
FOM	Figures of Merit
FRET	Fluorescence Resonance Energy Transfer
g-C_3_N_4_	Graphitic Carbon Nitride
Gd_2_O_3_	Gadolinium Oxide
Ge	Germanium
hBN	hexagonal Boron Nitride
IL	Interleukins
InP	Indium Phosphide
IO-Au	Iron oxide Gold
IR	Infrared Radiation
LEDs	Light Emitting Diodes
LOD	Limit of Detection
LSCI	Laser Speckle Contrast Imaging
LSPR	Localized Surface Plasmon Resonance
MgO	Magnesium Oxide
MMP	Matrix metalloproteinases
MNP	Metal Nanoparticles
MNP-GRP	MNP modified Graphene
MO	Magneto optical
MRI	Magnetic Resonance Imaging
NiO	Nickel Oxide
NIR	Near-Infrared Radiation
OCT	Optical Coherence Tomography
OFETs	Organic field-effect transistors
OLEDs	Organic light-emitting diodes
OPDs	Organic photodetectors
OPVs	Organic photovoltaic cells
PAI	Photoacoustic Imaging
PAT	Photoacoustic Tomography
PCCT	Photon Counting Computed Tomography
PD	Photodiode
PDs	Photodetectors
PEG	Polyethylene Glycol
PF	Polyfluorene
PFU/ML	Plaque-Forming units per milliliter
PLCL	Poly(l-lactide-co-ε-caprolactone)
PMICs	Patient Monitoring and Intervention Centers
P-NM	Plasmonic Nanomaterials
PPP	Polyparaphenylene
PPV	Polyparaphenylenevinylene
PRET	Plasmon Resonance Energy Transfer
PTFE	Polytetrafluoroethylene
QDs	Quantum dots
RIU	Refractive Index Unit
SERS	Surface Enhanced Raman Scattering
Si	Silicon
SiO_2_	Silicon dioxide
SNR	Signal-to-Noise Ratio
SPIO	Superparamagnetic iron oxide nanoparticles
ssDNA	Single Stranded Deoxyribose Nucleic Acid
TB	Tuberculosis
TiO_2_	Titanium Oxide
UV	Ultraviolet
ZnO	Zinc Oxide
